# Novel Contribution of Secreted Amyloid-β Precursor Protein to White Matter Brain Enlargement in Autism Spectrum Disorder

**DOI:** 10.3389/fpsyt.2019.00165

**Published:** 2019-04-10

**Authors:** Deborah K. Sokol, Bryan Maloney, Cara J. Westmark, Debomoy K. Lahiri

**Affiliations:** ^1^Pediatrics Section, Department of Neurology, Indiana University School of Medicine, Indianapolis, IN, United States; ^2^Indiana Alzheimers Disease Center, Department of Psychiatry, Stark Neuroscience Research Institute, Indiana University School of Medicine, Indianapolis, IN, United States; ^3^Department of Neurology, University of Wisconsin, Madison, WI, United States; ^4^Department of Medical and Molecular Genetics, Indiana University School of Medicine, Indianapolis, IN, United States

**Keywords:** amyloid, anabolic, brain overgrowth, biomarker, comorbidity, metabolites, neurodevelopmental, secretase

## Abstract

The most replicated neuroanatomical finding in autism is the tendency toward brain overgrowth, especially in younger children. Research shows that both gray and white matter are enlarged. Proposed mechanisms underlying brain enlargement include abnormal inflammatory and neurotrophic signals that lead to excessive, aberrant dendritic connectivity via disrupted pruning and cell adhesion, and enlargement of white matter due to excessive gliogenesis and increased myelination. Amyloid-β protein precursor (βAPP) and its metabolites, more commonly associated with Alzheimer's disease (AD), are also dysregulated in autism plasma and brain tissue samples. This review highlights findings that demonstrate how one βAPP metabolite, secreted APPα, and the ADAM family α-secretases, may lead to increased brain matter, with emphasis on increased white matter as seen in autism. sAPPα and the ADAM family α-secretases contribute to the anabolic, non-amyloidogenic pathway, which is in contrast to the amyloid (catabolic) pathway known to contribute to Alzheimer disease. The non-amyloidogenic pathway could produce brain enlargement via genetic mechanisms affecting mRNA translation and polygenic factors that converge on molecular pathways (mitogen-activated protein kinase/MAPK and mechanistic target of rapamycin/mTOR), promoting neuroinflammation. A novel mechanism linking the non-amyloidogenic pathway to white matter enlargement is proposed: α-secretase and/or sAPPα, activated by ERK receptor signaling activates P13K/AKt/mTOR and then Rho GTPases favoring myelination via oligodendrocyte progenitor cell (OPC) activation of cofilin. Applying known pathways in AD to autism should allow further understanding and provide options for new drug targets.

## Introduction

Autism or Autistic Syndrome Disorder (ASD) is characterized by lack in social communication and interaction and by restricted/repetitive patterns of interests, behaviors or activities (DSM-5) (1). It is considered the fastest growing neurodevelopmental disability. ASD prevalence estimates from the Centers for Disease Control (CDC) in 2014 were 1 in 68 children, which was up 30% from that reported in 2008, and more than double the frequency from 2000 (2). Autism affects more boys than girls. There is no agreed upon biomarker for ASD, however, the most replicated neuroanatomical finding is the tendency for heads and brains to be large, especially in young ASD subjects (3–7). Both macrocephaly—enlarged heads, as measured by occipital-frontal circumference (OFC), and megalencephaly, or enlarged brains, as measured post mortem or by MRI, are reported in autism. It is possible to have macrocephaly without megalencephaly. Unfortunately, the two are at times confused in the literature. Megalencephaly is increased growth of cerebral structures related to anomalies during brain development or because of postnatal abnormal events that cause excessive cerebral growth. On the other hand, macrocephaly, or simple increased head circumference, is linked to various events that are not necessarily neurological, including anomalies of bone skull structures, subdural fluid collections, hydrocephalus, intracranial masses, and arteriovenous malformations (8). However, only 20% of individuals with autism have enlarged brains (9), and the association between macrocephaly and core features of autism remains weak. This opens the door to entertain novel associations or apply knowledge from better-developed fields such as Alzheimer Disease (AD) to the study of autism.

AD comprises up to 80% of all dementias, affects 1 in 10 individuals over the age of 65, and affects more women than men (10). The amyloid hypothesis predicts clinical disease associated with amyloid-β loaded plaques resulting in brain atrophy in individuals with AD. Amyloid-β protein precursor (βAPP) and its metabolites are dysregulated in autism (11–15). βAPP is a large membrane spanning glycoprotein with a long extracellular N-terminus, a transmembrane region and an intracellular C-terminus, the βAPP intracellular domain [AICD; (16)]. After translation in the endoplasmic reticulum, APP undergoes post-translational modification in the Golgi complex before it travels to the cell membrane (17). The mature protein undergoes proteolytic cleavage by a combination of secretase enzymes. β- and γ-secretases generate amyloid-β (Aβ) peptides, primarily Aβ-40 and 42, found in the cerebral amyloid plaques of AD along with secreted APPβ (sAPPβ). α- and γ-secretase produce sAPPα, which is generally considered neurotrophic and neuroprotective, as well as AICD and p3 peptide. Under normal conditions, a small fraction of secreted βAPP is sAPPβ, produced via the amyloidogenic pathway. The majority of secreted product consists of sAPPα, produced constitutively via the non-amyloidogenic pathway. In AD, Aβ peptide is secreted at high levels and accumulates as neurotoxic extracellular aggregates. βAPP localizes to somatodendritic, axonal and the presynaptic active zones (18). βAPP expression is upregulated during inflammation (19) and is activated by proinflammatory cytokines and astrocytes (17, 20), so that APP may be involved in the acute phase protein response to immune stress. In synopsis, the localization of βAPP at dendritic synapses, its role in cell adhesion, its interaction with post-translational pathways and in transcription suggests that βAPP metabolites may play a pivotal role in the development of autism, and its single gene model kin, Fragile X Syndrome (FXS) (21, 22).

In this article we demonstrate how βAPP, particularly the non-amyloidogeneic pathway involving sAPPα contributes to the following mechanisms underlying excessive brain growth in autism: neuroinflammation, including cytokine activation, translational pathways, and increased myelination. These mechanisms were chosen as they are important to βAPP processing in AD, and serve to demonstrate catabolic vs. anabolic forces underlying these disorders.

In two independent studies (11, 21), our group has reported higher sAPPα levels and lower Aβ peptide levels in plasma of children with autism (*n* = 26). The same pattern was seen in autism temporal lobe brain tissue (*n* = 7) (21) of children with autism. The sAPPα results have been replicated by an independent laboratory in autism serum (15) and brain tissue (23). The finding of increased sAPPα also has been reported via confocal microscopy of brain tissue from groups of children with duplication 15q11.2-q13 and idiopathic autism (24). We have found increased levels of sAPPα, βAPP, and Aβ plasma markers in children with FXS (*n* = 12) compared to those with autism (*n* = 11) and typical development (*n* = 18), and in brain tissue from individuals with FXS [*n* = 3; (21)]. One of the purported α-secretase enzymes that cleaves βAPP and generates sAPPα (25), A Disintegrin and Metalloproteinase Domain 17 (ADAM17) also known as tumor necrosis factor-α converting enzyme (TACE), increases in autism brain tissue (21). ADAM17 is a member of the adamalysin family sheddases, and together with ADAMs 9 and 10, cleaves APP at the alpha-secretase site (26). Further, ADAM17 is pro-inflammatory, as it releases the cytokine tumor necrosis factor-α (TNF-α) during inflammation (27). ADAM17 and ADAM10 are essential for development of oligodendrocytes (28, 29), which produce myelin within the CNS. Several studies in the field point to a connection between differences in βAPP processing and autism (Table 1).

**Table 1:** Studies linking βAPP with autism.

**Study**	**Subjects/materials**	**Finding**
Lahiri et al. (30)	Brain samples from TD, AD, ASD, and FXS human subjects	Elevated βAPP and Aβ in FXS and AD, reduced Aβ and elevated sAPPα in ASD subjects, all vs. age-matched controls.
Ray et al. (21)	Plasma from TD, ASD, and FXS human subjects	Aβ and sAPPβ levels reduced and sAPPα elevated in ASD while Aβ and βAPP elevated in FXS, all vs. controls.
Ray et al. (13)	Plasma from TD, mild/moderate ASD, severe ASD human subjects	Aβ and sAPPβ levels reduced and sAPPα elevated in severe but not mild/moderate ASD vs. controls
Sokol et al. (11)	Plasma from TD, mild/moderate ASD, severe ASD human subjects	Aβ40 and Aβ42 levels diminished according to severity of autism (more severe → lower levels). Secreted APP increased alongside autism severity.
Bailey (15)	Whole blood samples from autism subjects and age-matched TD controls, human umbilical cord blood	sAPPα elevated in whole blood and plasma of autistic children. 7% of undiagnosed cord blood samples had elevated sAPPα
Wegiel et al. (24)	Brain samples from TD, ASD, and dup(15) human subjects	dup(15) subjects accumulated high levels of p3 peptide. ASD subjects had intermediate accumulation of P3, while controls had lowest levels of accumulated p3.
Bailey et al. (31)	Blood from human sAPPα overexpressing mice	sAPPα elevation corresponded to elevated CD8^+^ T cells and decreased effector memory T cells. Multiple signal and cytokine levels were also perturbed.

In total, these findings support the “Anabolic Hypothesis of βAPP in Autism” (14, 32), which postulates that sAPPα contributes to neuronal and glial overgrowth in the brain resulting in neuronal interference contributing to autism symptoms. One direct mechanism has been reported by Westmark and Malter (33) who found that βAPP is regulated via Fragile X Mental Retardation Protein (FMRP) via mGluR-5 in mouse models of FXS. When the FMRP “brake” is removed in translation, βAPP mRNA is increased. Of note, children with FXS often display macrocephaly, with a predominance of white matter (34). βAPP and metabolites play an anabolic role in other translation regulating pathways such as Mammalian target of rapamycin [mTOR; (35)], Ras small GTPase/Extracellular signal regulated kinase [Ras/ERK; (36, 37)] and phosphoinositol 3 kinase/mammalian target of rapamycin [P13k/mTOR; (37, 38)]. This sets the stage for the discussion of gray and white matter enlargement in autism, with the potential contribution from βAPP metabolites.

## Normal Brain Growth

Mammalian brain cells include neurons and glia. Neuron cell bodies and dendrites form the outer gray matter, with axons projecting downward into the subcortical white matter. Brain size depends both on the size of the cerebral cortex or gray matter and the underlying white matter (39). The volume of gray cortex is determined by cortical thickness, and cortical surface area, believed to be genetically independent (40). One model to explain cortical thickness and surface area pertains to radial cortical columns (41). Radial cortical columns depend on the number of neural stem cells that symmetrically divide in the ventricular zone before neurogenesis (39). The growth of progenitor cells predicts the number of neurons within the radial columns (42). Cortical thickness is predicted by the number of cells within the columns, whereas surface area is predicted by the number of cortical columns (41). The white matter comprises over half the human brain, in greater proportion than other animals (43). Intensely studied in demyelinating conditions such as multiple sclerosis, myelin, and white matter structure is dynamic, regulated by impulse activity, and essential to cognitive function (5, 43–45). Besides neuronal axons, white matter is composed of glia. Glia are of three types including: (1) astrocytes, which outnumber neurons five to one (46), contribute to synaptic transmission, neuronal processing (46) and form the blood brain barrier; (2) microglia, which act as innate immune cells providing host defense and tissue repair-these release cytokines, nitric oxide and various neurotrophic factors; and (3) oligodendrocytes which manufacture myelin sheaths for axonal insulation. The precursor to oligodendrocytes are oligodendrocyte precursor cells (OPCs), which are the only type of glia to receive synaptic input from neurons (44). OPCs produce neuron/glia antigen 2-NG2, which communicate directly with neurons and demonstrate the newly appreciated importance of glia, which seem to “talk back to neurons” (46). Glia are found in both gray and white matter, but there are more oligodendrocytes in white matter (47).

## Macrocephaly in Autism

### Anatomical Findings

In 1943, Leo Kanner (48) observed that some autistic children have large heads. Subsequent observations showed a strong trend toward enlarged OFC in autism, particularly in younger subjects (3, 49–51). However, individual reports must be evaluated carefully, since evaluations based on standard growth curves can give drastically different results depending on the specific curve. For example, a survey of over 75,000 neonatal OFC from infants in a single US-based primary care network (PCN) found macrocephaly in 8.6% according to CDC curves, in 14% according to the World Health Organization (WHO) curves, and in 5.1% according to National Center of Health Statistics (NCHS) curves. If the norm was the PCN's own distribution, only 4.4% had OFC > 95th percentile (52). Studies that compare autistic and non-autistic groups directly should be considered more reliable than other studies (51, 53, 54). Indeed, use of population norms may have introduced systematic age-dependent bias into nearly 83% of the field's studies on head circumference (HC) vs. autism (54). The effect of autism vs. other known influences on head size also may have been chronically over-stated. A systematic review of ~400,000 HC measures on ~75,000 children found that when models also included covariates known to strongly influence HC regardless of autism, such as parental height and parental HC, the covariates played a large role in child HC even when the parents were not themselves autistic. In addition, the best-replicated aspects of early brain overgrowth across studies were more reflective of biases in HC norms than specific ASD traits or biomarkers (54). In a specific study, once gender, age, height, weight, and parental contribution were taken into account, the contribution of autism came to an overall effect of +2 millimeters. This small effect was, however, significant (53).

Nevertheless, in properly designed, control-matched studies, autism still associates with macrocephaly, even though most autistic children are not macrocephalic. Compared to typical development, autistic brains show a growth spurt shortly after birth, continuing in the first year of life, suggesting postnatal pathology. Neuropathological evidence of brain overgrowth include increased brain size and weight (55–57) and the early migration of pyramidal cells with more numerous, smaller, and less compact minicolumns (58–60). Minicolumns, described as vertical groups of large neurons flanked by cell-sparse surroundings, would require the growing support of short-range fibers that make up the white matter without affecting neuron density, as the brain grows larger (61). Large brains have been noted in autism, particularly in children <4 years of age, in cross sectional MR brain volumetry studies (3, 4, 62–64). Autism investigators have reported increases in total cerebral volume (64–66), cortical thickness (54), cortical surface area (39, 67), regional gray (60, 65, 68), and white matter (44, 69). A recent volumetric MRI longitudinal study of 200 children with autism identified 15% of this sample as boys with large heads disproportionate to height or “disproportionate megalencephaly” (70). Further study of this megalencephalic cohort revealed increased surface area but not increased cortical thickness (39), as well as increased white matter of the brain (70).

At young ages (2–6 years), brain enlargement occurs seemingly coincidental with the display of autistic symptoms (62, 69). This is followed by abnormal decline in brain growth by 12–16 years (71). This raises the question if genetic growth factors/neuroinflammation can cause early brain enlargement resulting in later destruction. Further, brain enlargement appears to be greatest in the frontal and temporal cortices and amygdala and least in the occipital regions. This corresponds to known deficits in autism in social skills (frontal lobes), language and communication (temporal lobes), and emotional control (amygdala) with sparing of the visual spatial abilities (occipital lobe) (71).

Few studies to date have undertaken cellular analysis to determine the neural underpinnings of brain overgrowth in autism. For example, an increased number of neurons in autism prefrontal gray matter could contribute to brain overgrowth (71). This study raises the possibility that a prenatal cause of autism as neurogenesis is complete before birth in prefrontal cortex and all of cerebral cortex (72) However, no similar studies have been performed on white matter. Myelin and white matter continue to develop until the third decade, which involves processes other than increased number of neurons, such as gliogenesis and myelination, as shown by mutations of the phosphatase and tensin homolog (PTEN) gene associated with autism and macrocephaly (73), as discussed below.

### Functional Correlations of Increased Brain Growth in Autism

The functional correlates of enlarged brains in autism have eluded researchers. Early autism studies showed no association between macrocephaly and clinical function such as IQ or seizure frequency (9, 74), whereas in others, macrocephaly was associated with higher function (50, 75). However, IQ and seizure frequency are *not* core traits of autism. Instead, it may be that macrocephaly associates more strongly with *specific endophenotypes within the autism spectrum*, rather than autism in general. For example, within autistic boys (but not girls), greater HC associated with higher (more dysfunctional) scores in the Autism Diagnostic Interview-Revised (ADI-R) social algorithm scale (9). Further evidence that HC may correspond to different autism subtypes is that the HC of boys with regressive autism was significantly greater than non-autistic boys or boys with non-regressive autism. No HC differences were found for girls vs. regression (64).

Regional brain enlargement also is associated with higher function: enlarged amygdala in young children with autism, ages 2–4, was associated with joint attention ability, a core clinical feature of autism (76), and the larger volume of corpus callosum in younger preschool children was associated with lower severity of autism core symptoms (77). However, enlarged amygdala in autism does not necessarily entail macrocephaly (78). In a more comprehensive study of adults with autism with average intelligence (68), both cortical thickness and voxel based volume measurements identified enlarged gray matter regions that are associated with autism core features including the inferior frontal cortex, superior temporal sulcus, cingulate gyrus, middle occipital gyrus, fusiform gyrus, and inferior parietal lobule, which are associated with social abilities; superior temporal sulcus and inferior frontal gyrus, which are associated with communication; and orbital frontal gyrus and anterior cingulate gyrus, which are associated with repetitive behaviors. In particular, boys with disproportionate megalencephaly and autism had more severe disabilities, and a poorer prognosis (65), language deficits (79), and showed more regression (64). Large brains in autism may be responsible for more global deficits such as weak coherence theory, the ability to understand context or the “big picture,” instead of more discrete autism core features (44).

## Mechanisms of Brain Overgrowth in Autism

### Mechanisms of Increased Cortical Gray Matter in Autism

As stated above, cortical thickness is predicted by the number of neurons within the radial cortical columns, whereas surface area is predicted by the number of cortical columns (41). Casanova et al. (58, 60) have reported reduction in minicolumn width and neuropil spacing in layer III neocortex reflecting an increase in minicolumns in autism. β-catenin and caspase 3/9 proteins have been implicated in cortical surface area. Transgenic mice expressing β-catenin develop an increased number of progenitor cells in the ventricular zone which leads to more cortical radial columns and increased surface area (80). Caspase 3/9 mutations result in decreased apoptosis of progenitor and radial glial cells, which increase surface area (81). Although this paper proposes a relationship between anabolic βAPP and increased white matter in autism, there are mechanisms potentially linking βAPP to increased gray matter as well. For example, βAPP shows reciprocal regulation of the anabolic Wnt/β-catenin signaling pathway, and βAPP physically interacts with β-catenin (82). Furthermore, βAPP decreases apoptosis in part by decreasing Caspase 3/9 by means of the phosphoinositide 3-kinase and Protein kinase B (PI3K/AKT) pathway in AML1-ETO positive (AE) leukemia (83). As described below, the contribution of βAPP to the PI3K/AKT pathway may be involved in white matter growth in autism.

### Mechanisms of Increased Cortical White Matter in Autism

Autism has been described as a disorder of brain connectivity (84) and therefore the white matter, subjacent to gray matter cortex, which connects discreet cortical regions, is a region of interest. Increased white matter in autism exists in whole brain and cerebellum (62), in radiate outer zones (6), and in specific brain regions (85–88). Herbert (44) observed larger cerebral white matter volume in children with autism as well as in children with developmental language disorders. This was replicated in a larger sample of children with autism compared to control groups of children with benign macrocephaly and reading disability (89). Newer imaging techniques such as diffusion tensor imaging (DTI) have uncovered differences in white matter tract microstructure in autism (90–92). A review of cross sectional DTI in autism showed results consistent with an abundance of local, short white matter connections within lobes (e.g., temporal lobes) and reduced long distance connections (corpus callosum) between lobes (93, 94). Although white matter tracts appear disrupted in autism, the neuropathology underlying the disruption is not clear from DTI studies (93). Recent review of DTI longitudinal studies showed increased fractional anisotropy, a measure of white matter integrity, at 6 months of age, remaining atypical until 24 months of age and a reduced rate of white matter growth in late childhood-early adolescence in the left parietal, left occipital and bilateral temporal lobes (88). Hypermetabolism, as measured by fluorodeoxyglucose positron emission tomography (PET), was found for white matter within the internal capsule, corpus callosum, frontal, and temporal lobes of young adult subjects with autism, similar to patients with schizophrenia (95). This was explained by disruption of myelination (95). Altogether these results suggest at least a transient increase in white matter volume that may recede over time.

We propose that βAPP metabolites may contribute to mechanisms of increased white matter in autism. Thus, we discuss neuroinflammation, cytokine release, translation pathways and increased myelination. In contrast to increased neurons found in autism gray matter (71), neuroinflammation [including astrogliosis and microgliosis; (96, 97)], along with increased myelination, may contribute more to the white matter enlargement in autism (43, 44, 94). Genetic mechanisms proposed to cause macrocephaly and autistic traits include genes affecting mRNA translation pathways (98). Many factors, including neuroinflammation and polygenic factors may converge on molecular pathways (mitogen-activated protein kinase -MAPK and mechanistic target of rapamycin- mTOR), which could explain significant features of ASD, including white matter enlargement. In this review, we propose that secreted amyloid precursor protein metabolites, particularly sAPPα, contribute to ASD white matter enlargement, ultimately through MAPK and protein translation pathways that may stimulate increased gliosis and/or myelination This is a novel application of pathways developed for AD which may invite new understanding and treatment for autism.

## Neuroinflammatory Effects

### Neuroinflammation in Autism

How can neuroinflammation contribute to brain overgrowth in autism? Up until 2005, it had been assumed that autism did not involve an inflammatory process, as there were no reports of gliosis or replicable inflammation in neuropathologic studies or brain MRI (44). By using immunochemistry and cytokine protein arrays, Vargas first showed an increase in CSF level cytokines (macrophage chemoattractant protein-1 and tumor growth factor β1) and microglia-astroglial activation (increased glial fibrillary acidic protein-GFAP) in the medial frontal gyrus and cerebellum in children and adults with autism (96). Further studies showed microglial pathology in autism (60, 99, 100). Specific decrease in ramified microglia (said to underlie deficits in synaptic pruning) and increase in primed microglia (associated with chronic inflammation) were found in autistic gray and white matter (97). Neuroinflammation in autism appears to be similar to AD (44) showing microscopic but not MRI signs of inflammation. Confocal microscopy study of children with idiopathic autism and dup15q11.2-q13 revealed that p3 peptide (a neurotrophic, non-amyloidogenic βAPP processing product) aggregates in astrocytes and microglia (24). Using confocal microscopy, specific antibodies identified higher equivalents of sAPPα in gray and white matter structures for dup15q11.2-q13, followed by idiopathic autism, followed by typical controls.

Reports exist of peripheral blood abnormalities involving T-cell, B-cell, autoantibody production, and increased pro-inflammatory cytokines (101), but these studies were limited by cross sectional design, small sample size, subjects of different ages, and lack of standardized diagnosis. A recent longitudinal study of serum (with typically developing controls) and cerebrospinal fluid (no controls) from young children with autism (ages 2–8) surprisingly found no evidence of immune mediators supportive of active systemic inflammation in autism (102). Only “modulators of immune function,” Epidermal Growth Factor (EGF) and soluble CD40 ligand were increased in autism serum (102). The authors concluded that this was due to a “genetically determined growth or immune-modulatory dysregulation” rather than an active systemic inflammatory response (102). The following CSF immune mediators were elevated: FLT3L, IL-15, CX3CL1, CXCL8, and CCL2, and their role interpreted as homeostatic in support of microglia rather than an adaptive neuroinflammatory response. The biggest finding was the lack of overlap between peripheral and cerebrospinal markers and of serum markers over time, demonstrating no reliable peripheral biomarker (102).

Given the possibility of a genetically upregulated immunity in autism, how would this produce brain overgrowth? Herbert (44) suggests that white matter volume increase is due to cell swelling/tissue volume increase as a result of activated microglia and astroglia, or via a compensatory increase in vascularization. Neuroinflammation could trigger cytokine or chemokine release (44), as identified by Pardo (102), or other secondary signaling pathways such as mTor-PI3-Akt. Alternatively, neuroinflammation could lead to increased excitotoxicity with increased oligodendrocyte activity leading to an increase in myelination. Persistent, chronic neuroinflammation over time could then lead to cell death. This would explain the longitudinal changes of overgrowth followed by decrease in growth in autism brain MRI studies. Altogether, this sets the stage for the role βAPP metabolites may play in neuroinflammation, cytokine activation, and excessive myelination in autism.

### Neuroinflammation and βAPP

AD is a neurodegenerative disorder characterized by ongoing, permanent memory impairment and dementia. It is construed as a multifactorial disease affected by genetic and environmental factors. Neuroinflammation, vascular compromise and free radical damage lead to the abnormal accumulation of cerebral Aβ peptide and ultimately death (103). Alpha secretase, now believed to be ADAM10 or ADAM17, constitutively cleaves βAPP to produce sAPPα, p3 peptide, and AICD along the non-amyloidogenic (neurotrophic) pathway. The β-amyloid hypothesis states that improper cleavage yields Aβ peptides found in the cerebral amyloid plaques of AD. Early in the disease, microglia are protective, increasing the phagocytic clearance of Aβ peptides. However, overtime, microglia become more catabolic producing proinflammatory cytokines IL-1 and TNF-α with the latter being more detrimental, eventually leading to brain atrophy seen in AD. Inflammatory mediators, in turn, cause more Aβ production (104). Evidence supports sAPPα activity on microglial cells, activating release of IL-1 (105), glutamate and inflammatory markers (106, 107). Treatment of neural stem cells with recombinant sAPPα led to increased astrogliogenesis (108). Treatment with sAPPα differentiates neuronal progenitor cells into astroglia (109).

ADAM17is one of the alpha secretases that is active in AD. It is found in higher levels in the CSF of patients with AD and its precursor Mild Cognitive Impairment (MCI) (110). ADAM-17 positive neurons are often found together with amyloid plaques in AD brains, suggestive that ADAM17 is involved in AD pathogenesis (111). ADAM17 produces neurotrophic sAPPα following the non-amyloidogenic pathway, but also contributes to the neuroinflammation in AD microglial activation (112). ADAM17, therefore, has the unique role of contributing to or preventing AD (113).

ADAM17 promotes cellular growth and acts as an extracellular physiological convertase, shedding proteins other than APP, including the EGF family of growth factors. These include TGF-α and its receptor EGF (114). ADAM17 contributes to generation and maturation of TNF-α, EGR, and some cell adhesion molecules [CAMS; (115)]. ADAM17 and ADAM10 cleave Notch proteins to induce Notch signaling (116). Further, acting anabolically, ADAM17 (and its kin, ADAM10) (mal) function in the pathogenesis of cancers, rheumatoid arthritis (117), and spinal cord injury (118). Besides converting EGF (114), the peripheral immune mediator found in the Pardo 2017 study of children with autism (102), ADAM17 converts the other cytokine mediators of autism found in the Pardo study: IL-15 (119), FLT3 (120), CX3CL1(121), and CXCL8 (122).

### Key Translation Signaling Pathways in Inflammation

Key biochemical pathways involved in the immune response are of great interest as they also play a central role in growth and metabolism and may be involved in brain overgrowth in autism. mTOR and MAPK pathways regulate immune function (123). mTOR is composed of two structures, mTOR complex 1 (mTORC1) and mTOR complex 2 (mTORC2). More is known about mTORC1 and its immediately upstream regulator Ras homolog enriched in brain (RHEB). RHEB is controlled by GTPase-activating protein (GAP), which involves the tuberous sclerosis complex 1 (TSC1 and TSC2). When this complex is phosphorylated by Akt or ERK1/2 (through phosphatidylinositol 3 kinase), this inhibits GAP activity so that RHEB is active, which in turn activates mTORC1. Several cytokine and growth factors activate mTOR including CD28, IL-1, IL-2, IL-4, and IL-12. mTOR is involved in T-cell trafficking as upon recognition of their antigen, CD8^+^ T cells activate mTOR and switch to anabolism. Upon resolution of the infection, the antigen-specific CD8^+^ cells contract and mTOR activity is decreased. mTOR promotes differentiation, activation, and function in T-cells, B-cells, and antigen presenting cells (123). The mTOR anabolic biochemical pathway is of great interest in autism (124) and in macrocephaly and autism (125). mTOR deregulation also exists in AD (35). Evidence supports βAPP's role in activating T-lymphocytes. For example, transcription, translation and secretion of βAPP have been induced via T-cell mitogen stimulation of blood leukocytes (126). sAPPα modulation of the immune system has been studied in an overproducing sAPPα mouse model (31). These mice showed increased levels of CD8 T-cells and decreased memory T-cells suggesting that sAPPα activates immunity.

The MAP kinase cascade is composed of three major groups: the p38 MAP kinases, the *c*-Jun NH2-terminal kinases (JNK) and the extracellular signal-regulated protein kinases (ERK). MAP kinases are important in lymphocyte development. Activation of MAP kinases p38 and JNK produces inflammatory cytokines including TNFα, IL-1, and IL-12. In turn, TNFα and IL-1 activate p38 and JNK MAP kinases. It has been shown that JNK and p38 activation acts catabolically, leading to apoptosis in AD (127). ERK regulates T cell activation and differentiation (128). The growth factor signaling properties of the MAPK pathways act anabolically, and are of interest in autism (124, 129, 130), with convergence of the MAPK pathway with calcium signaling pathways reported as major contributors to autism pathophysiology in a large gene set enrichment analysis (131). We speculate that TNFα, elevated in autism, favors increased p38 and JNK resulting in increased brain matter, while decreased TNFα promotes apoptosis via p38 and JNK in AD.

## Regulatory Factors

### Purported mTOR Regulation in Autism and AD

Neurotrophins, including nerve growth factor (NGF) and brain-derived neurotrophic factor (BDNF) bind to and activate tropomyosin-related kinases (TrkA, TrkB, or Trk C) which in turn activate PI3K, phospholipase C (PLC), ERK1/2, and mTOR (132). Neurotrophin signaling at multiple levels (epigenetic, transcriptional, post-transcriptional, and post-translational) determines cell fate, axonal and dendritic growth and pruning, and the connection of neural networks (124). Abnormalities in neurotrophins likewise exist in autism and AD (132).

mTOR is involved in promoting growth by means of protein synthesis, actin cytoskeletal dynamics, energy homeostasis and metabolism (124). Gene defects associated with upstream regulators of mTOR (TSC1, TSC2, and PTEN), are associated with autism and in many cases, macrocephaly. Constitutive activation of upstream PI3K, Akt, and Ras, is anabolic, and associates with macrocephaly, neuronal hypertrophy, increased soma size and dendritic complexity of neurons (133). Products of mTOR that contribute to translation such as p70 ribosomal S6 kinase 1 (Sgk1) and eukaryotic translation initiation factor 4 E-binding proteins (4E-BPs) may play a role in autism (98, 134). Over expression of eIF4E associates with an increase in complex formation, long-term synaptic plasticity, increased dendritic spine density, and behaviors similar to autism (135, 136). 4E-BPs provide a brake on eukaryotic translation initiative factor 4E (EIF4E) which is a primary effector through which proteins are synthesized. Phosphorylation of 4EBP by mTORC1 stops this inhibition favoring EIF4E, and anabolic protein synthesis (135). Huber's view, based on research with FXS, supports a model where FMRP represses a PI3K enhancer (PIKE) so that mTOR is inhibited under basal conditions (137). Lack of FMRP in FXS (and in some individuals with autism), would also favor PIKE activation of mTOR and downstream Rho GTPases including Rac1 and cofilin that in turn would cause actin disassembly and myelination (138). Recently, several studies have identified a catabolic role of mTOR, and the upstream and downstream components of its signaling, in the pathogenesis and progression of AD (35). In some models, activation of mTOR is interpreted as preventing autophagy of Aβ in the lysosomes. However, treatment of AD with mTOR inhibitor rapamycin was not recommended, as rapamycin decreases ADAM10, an alpha secretase that may promote the protective, anabolic, non-amyloidogenic pathway.

Myelination is metabolically demanding, and therefore, the mTOR pathway, particularly the mTORC1 hub coordinating cell metabolism, is a key signal for myelination (139). Besides its main function as a regulator of mRNA translation, mTORC1 activates lipid synthesis. Lipid is a major component of myelin. Further, mTORC1 appears to coordinate protein and lipid synthesis to make the membrane. It differentiates oligodendrocytes from OCPs. Interestingly, hyperactivation of oligodendrocytes after disruption of the TSC complex by deletion of TSC1 effects *hypomyelination* in the CNS and PNS (140, 141). Hypomyelination and decreased oligodendrocytes were also reported after deletion of TSC2 (142). These changes were unexpected and not in keeping with the clinical picture of macrocephaly and often megalencephaly with white matter increases and tubers seen in tuberous sclerosis caused by TSC1 or TSC2 deletions, a single gene kin to autism. Therefore, other factors besides overactivation of mTOR must lead to excessive white matter in autism. Upstream from mTOR, Akt-1 is phosphorylated by PI3K in response to growth factors such as Neuregulin 1, insulin growth factors and steroids that promote myelination (143) The insulin growth factor 1 (IGF-1), when phosphorylated, stimulates the P13K/Akt and MAPK pathways and has been implicated in AD (144). IGF-1 acts anabolically, stimulating α-secretase and reducing β secretase that has been shown to decrease Aβ formation (145). In contrast, Aβ acts catabolically, resulting in over-expression of PTEN which leads to deactivation of P13K/Akt (146). Deletion of PTEN activates the P13K/Akt pathway which results in increased myelination, especially within the corpus callosum. Individuals with PTEN syndrome show macrocephaly and autism (73). It appears that there are several pathways that lead to myelination, perhaps via activation of the Rho family proteins that in turn activate myelination through sAPPα/α-secretases.

### Purported MAPK Regulation in Autism and AD

ERK 1/2, among the MAPK family members are involved with cellular growth, chemokines, oxidative stress, and cytokines. Single gene mutations associated with autistic-like syndromes can also cause ERK1/2 activation including Tuberous sclerosis, FXS, 16p11.2 and Neurofibromatosis. Macrocephaly and increased white matter are often seen in these conditions (98). However, of far greater interest to investigating the much larger proportion of idiopathic autism, this pathway can be activated by multiple stimuli, both internal and environmental. These include interleukin-1β (IL-1β), TNF-α, and EGF (147), and EGF receptor [EGFR; (148)], all which are elevated in autism. The ADAM proteins play critical roles in EGFR signaling (115, 122, 149). Several studies have found an association between MAPK signaling and autistic traits. Indeed, genes from the MAPK pathway were among the most frequent represented in a large gene set analysis in autism (131). This pathway has also been intensely researched in cancer studies, as derangement in MAPK signaling may be important in the development of cancer (150, 151). ERK and MAP kinase p38, which are activated in response to inflammation or stress signals, directly activates ADAM17 (152, 153). Sources of inflammatory stimulation of MAPK include infection by Gram-negative bacteria, and ensuing lipopolysaccharide (LPS) exposure. Several mechanistic studies of LPS exposure in model animals showed that LPS exposure induced both behavioral and neurological autism-like symptoms, and that this induction was stronger in male than female offspring (154–158). The MAPK pathway is less implicated in pathogenesis of AD, although JNK and P38 activation leads to apoptosis in AD (159). The neurogenic properties of this pathway in association with those of the non-amyloidogenic pathway have been studied and will be discussed below.

ERK1 and ERK2, downstream mediators of MAPK appear to control CNS myelin thickness after oligodendrocyte differentiation and initiation of myelin (160), as demonstrated by *in vivo* loss of function studies (160) and *in vivo* gain of ERK1/2 function studies (161). In the latter, two lines of transgenic mice with sustained activation of OPCs during early development produced transient over-proliferation of OPCs, but resulted in normal numbers of myelinating oligodendrocytes. This was interpreted as ERK 1/2 effecting a biphasic response-first an early expansion of OPC and a later promotion of myelin growth. Another MAPK regulator, P38, directs oligodendrocyte differentiation and myelination by way of gene transcription (162). BDNF increases OPC proliferation and development though the TrkB and MAPK pathway (163).

## Hypermyelination—a Source of Increased White Matter in Autism?

Having discussed how neuroinflammation, potentially causes brain tissue edema and increased perfusion and how activated signaling pathways associated with growth factors could contribute to activation of white matter, we will now turn to the potential contribution of myelination to increased white matter in autism.

Oligodendrocytes ensheath multiple neuronal axons with a lipid-rich myelin membrane. Myelin allows rapid synaptic transmission, provides metabolic support and reduces the cost of neuronal energy (164). There has been much effort to understand myelin formation in the hopes of promoting myelin repair for conditions such as multiple sclerosis. The belief that remyelination “depends on signals that are similar to those occurring in developmental myelination” (143), provides a window into the origins of myelination and allows speculation as to what myelinating processes may be in overdrive in autism. During development, myelinating oligodendrocytes are produced by OPCs within the subventricular zone (SVZ) of the germinal matrix in the cortex. OPCs then undergo migration and proliferation and extend through the entire nervous system. OPCs retain the ability to migrate and to travel within the CNS into adulthood where they continue to generate new oligodendrocytes routinely (165), and after demyelinating injury (166). In recent years it has been discovered that OPCs do more than just give rise to oligodendrocytes, as “they are found throughout the brain in numbers far greater than would be needed for that role” (46). OPCs are the only glia that receive synaptic input from neurons by way of an NG2 protein (28). ADAM10, one of the purported alpha secretases, cleaves this protein in response to neuronal network activity (28). It appears that NG2 cleavage functions to strengthen long-term potentiation (LTP) in the mouse somatosensory cortex, so, if not a growth factor, overexpression of ADAM10 may yield LTP aberration through OPC NG2 cleavage. OPC polarity and directional migration also appears to be under the control of NG2 glia (167), which features RhoA and Rac signaling. It is worth noting here that mTOR regulates cofilin through Rho family member Rac1, important in myelination (see below) and implicated in autism (137). OPC embryonic development is guided along endothelial cells by the powerful anabolic Wnt signaling (168), a pathway associated with autism (169), and regulated by the NOTCH canonical pathway (170) and βAPP (171). There appear to be several paths to increased myelination, and perhaps autism, should OPC N2 glial cells get excited.

Another view is that the consistent, abundant supply of OPCs in the brain may be required for developing novel motor skills (172). Myelination is a developmental process and can continue into the third decade of life (173). Myelination can change according to environmental experience (43). Early studies reported oligodendroctyes increase by 27–33% in the visual cortex of rats raise in enriched environments (174), and the number of myelinated axons within the corpus callosum increases in rats (175) and rhesus monkeys raised in enriched environments (176). Myelination of specific areas of the brain correlate with children's cognitive ability (177) and learning new tasks (178, 179). Indeed, activity dependent communication between axon and oligodendrocytes may cause increased oligodendrocyte production, and thicker and longer myelin on axons (164). However, too much stimulation may be deleterious. In rats, stress during late pregnancy causes hypermyelination in the offspring (180). Maternal IgG antibodies directed against fetal brain, considered immunologic stress, are elevated in mothers of and children with autism (181–183). These human maternal antibodies were injected into pregnant rhesus monkeys and their offspring followed for 2 years (184, 185). Their offspring showed subtle autistic behaviors; neuropathology revealed enlarged brains notable for increased frontal lobe white matter (184).

During OPC differentiation and myelination, OPC processes change from thin membrane extensions to multi-layered, lipid rich tubes ensheathing axons (164). The myelin sheath growth occurs in two steps. First, an actin network supports the leading edge clamping it between the axon and overlying oligodendrocyte. Second, actin disassembly allows the myelin membrane to spread around and along the axon (138). Actin is disassembled by cofilin and gelsolin family proteins. Myelin Basic Protein (MBP), an important component of CNS myelin, is necessary for myelin wrapping. MBP promotes myelination by releasing cofilin and gelsolin from the membrane and deactivating actin. The Rho1/Rac polarity of the OCP NG2 demonstrates how oligodendrocyte differentiation might be paired with axon wrapping (164). Pertinent to APP, MBP is a potent inhibitor of Aβ fibrillary assembly (186). Similar to how MBP functionally disassembles actin, the C-terminal of MBP_1−64_ binds to Aβ40 and Aβ42 peptides to inhibit fibril assembly. This would deter developing AD. These authors Kotarba et al. note that in human brain and in APP transgenic mice, Aβ peptides usually are not seen in MBP rich white matter. Several studies show increased levels of autoantibodies to MBP in the sera of children with autism (187, 188), implying that cerebral MBP levels may be high, although MBP has not been extensively studied in autism brain tissue. In AD brain tissue, MBD was degraded, suggesting destruction of white matter in AD (189). There is *in vitro* evidence that ADAM8 (not an alpha-secretase) cleaves MBP (190, 191), which may indicate an inflammatory reaction in AD (191). Therefore, reduction of MBP may lead to increased Aβ peptides and brain atrophy in AD, whereas increased MBP favors myelination and brain growth in autism. Could these mechanisms in autism confer protection from AD? This question recently has been discussed (32).

## ADAM Family α-Secretases

ADAM secretases perform many duties and play a role in CNS myelination. For many years, it was known that ADAM family members, particularly ADAM22, were important in peripheral system myelination (192), although, ADAM17 inhibited Schwann cell myelination (193). However, recently ADAM17 turned out to be “essential for oligodendrocyte development and CNS myelination” (29). ADAM17 modulated OPC cell cycle exit and oligodendrocyte lineage cell survival during subcortical white matter development in transgenic mice. ADAM17 accomplished this by shedding EGFR ligands and performed activation of oligodendrocytes during white matter development by EGFR signaling. EGFR overexpression in ADAM17-deficient OCPs restored cell survival and proliferation and subsequent myelination. Unlike other reports of diseased states, this study was distinguished by the analysis of oligodendrocytes during postnatal CNS myelination. The authors noted a divergence of ADAM17 function between the CNS and PNS (29).

## Notch

Notch signaling is essential for glial development and CNS myelination (164, 170). Notch receptors are cleaved intracellularly by secretases. In the CNS, Notch 1 receptor is expressed by oligodendrocytes. Studies show that Notch 1 is important for correct OPC temporal and spatial differentiation (164). Contactin-1, a ligand of Notch, may promote OPC differentiation within the expression of CNS myelin genes. The contactin family of Ig cell adhesion molecules harbor several members that have a genetic association with autism (194). Following the canonical pathway, ADAM10 cleaves NOTCH at the membrane which activates a piece that participates in transcription. Activation of NOTCH within the non-canonical pathway leads to OPC maturation and myelination. APP interacts with Notch receptors and the APP gamma secretase that produces the Aβ peptide is a Notch family member (195).

## The Case for sAPPα Directing Increased White Matter in Autism

Typical processing of βAPP greatly favors production of sAPPα over Aβ peptide. Could an increase in α-secretase activation overproduce myelin to the extent of inducing or exacerbating autism? As previously described, ADAM17 appears to act directly on oligodendrocyte development and CNS myelination and in oligodendrocyte regeneration (29, 149). *In vitro* α-secretase activation produces an increased number of mature oligodendrocytes and increased percentage of myelinated axons with short internodes (196). One clinical study of whole-exome sequencing in children with macrocephaly and/or autism showed a surprising 10/21 patients with likely pathogenic mutations along the PI3K/AKT-mTOR pathway (125). It is possible that similar underlying, closely related genetic mutations could push sAPPα into overdrive, resulting in autism.

**Figure 1:**
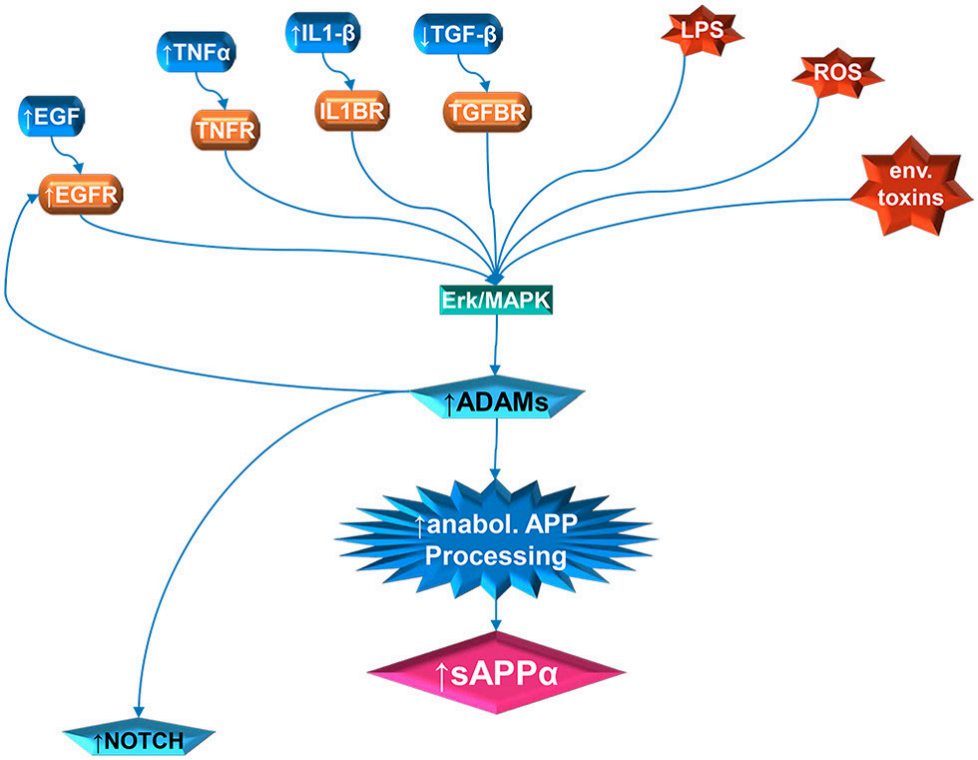
Multiple pathway stimulation of ADAM activity, leading to sAPPα proliferation and notch stimulation. The contribution of white matter overgrowth to autistic symptoms could begin with aberrant signaling of the ERK/MAPK pathway. Multiple extracellular signal molecules have disrupted levels in autism. This includes IL1β, EGF, and TNFα, which are elevated; and TGFβ, which is depressed. In addition, the receptor for EGF (EGFR) is elevated in autism. External stressors, such as LPS and reactive oxidizing species (ROS) produced by oxidative stress, have been implicated in autism. Activity of specific environmental toxins may exist but is still controversial. Once the ERK/MAPK pathway has, by whatever means, been perturbed, this can stimulate the ADAM proteins. The ADAMs not only cleave APP at the anabolic cleavage site, producing sAPPα, but are also necessary for EGFR signaling and stimulate NOTCH enzyme activity.

Increased sAPPα in autism could be a neuroinflammatory response with resultant upregulation of the non-amyloidogenic pathway. Bailey et al. (23) demonstrated increased sAPPα, neuroinflammatory GFAP and gliosis in transgenic mice designed to overexpress sAPPα in brain tissue. Upregulation of GFAP was correlated with elevations in Interleukin 6 (IL-6), gp130, and Notch1. The IL-6/gp130 pathway is considered anabolic and promotes axonal sprouting of neurons and activated astrocytes after entorhinal cortex lesion in rats (197). Furthermore, ADAM17 participates in the cleavage of inflammatory factors related to microglial activation (113) and in reaction to injury (118). A prenatal insult would also increase the expression of NMDA and glutamate receptors in the offspring. High glutamate receptors activate the ERK signaling cascade. Zeidan-Chulia et al. (198) proposed this model for autism after performing a focused microarray analysis of genes belonging to NOTCH, WNT and AD. They found upregulation of glutamate ionotropic receptor NMDA type subunit 1 (GRIN1), and MAP3K1, which activates the JNK and ERK pathways. Among their conclusions, they proposed that epigenetic stress could lead to increased NMDA receptors and increased calcium that would stimulate ERK-dependent α-secretase activity. Activation of ADAM17 by ERK (and P38 MAP kinase), for example, can activate EGF receptor signaling which leads to enhanced cell proliferation (152, 153). Higher levels of sAPPα would then activate the PI3K/Akt/mTOR pathway also resulting in aberrant brain growth. This model applies to brain cells in general, not just white matter. However, as described above, mTOR pathways favors myelination, except for the findings that disruption of upper mTOR pathways result in hypomyelination instead of hypermyelination (123). Therefore, other pathways such as activation of ERK1/2 may be needed to explain increased white matter in autism (Figure 1).

**Figure 2:**
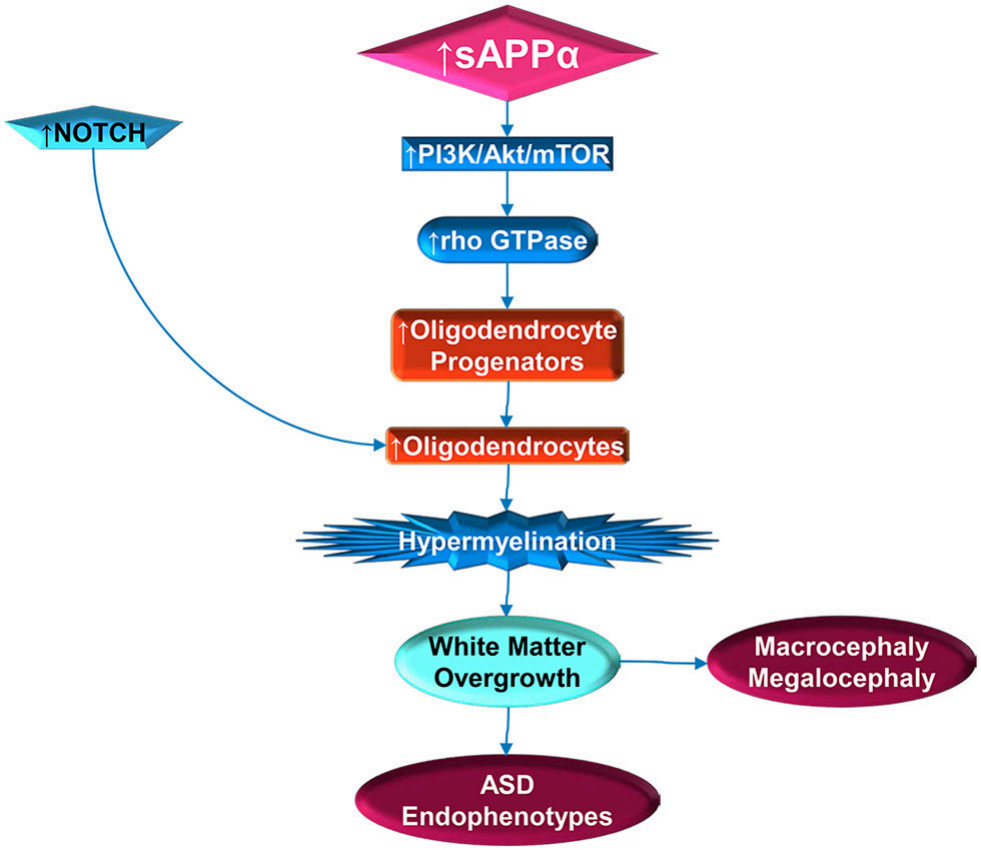
Stimulation of white matter overgrowth by elevated sAPPα (and Notch). Elevated sAPPα enhances PI3K/Act/mTOR pathway activity, which includes rho GTPase. The GTPase stimulates oligodendrocyte progenitors, which require NOTCH activity to mature into oligodendrocytes. Elevated oligodendrocytes would then contribute to hypermyelination, which would lead to white matter overgrowth. The white matter overgrowth would contribute to exacerbation of ASD endophenotypes, such as poor social functioning or regression. Incidentally, this would also be reflected by macrocephaly and megalocephaly.

The intersection of sAPPα, its ADAM family secretases and white matter expansion may be most convincingly proposed by merging the above mentioned models (137, 198) with the recent finding that the abundant OPC N2G is regulated by Rho GTPases (164). α-secretase, activated by ERK receptor signaling, in turn may activate PI3K/Akt/mTOR, and then Rho GTPases, which would favor OPC stimulation (Figure 2). Subsequent activation of cofilin with the disassembly of actin also favors myelination, again stimulated by α-secretase. Recent findings that netrin-1 appears to underlie OPC density and turnover (199), and that OPC migration along blood vessels is mediated by WNT signaling (168), further opens the door to APP regulation of myelination. APP regulates Netrin-1 mediated commissural axon outgrowth (200), and Wnt signaling is protective against Aβ peptide akin to the non-amyloidogenic pathway (201). Future study of α-secretase in relation to autism may enable novel treatments and avoid pitfalls in tested treatments for AD. One example would be to determine if ADAM17 antibody could reduce excessive brain growth and autistic symptoms, similarly to the recent success of the drug BAN2401 (202), an antibody that targets Aβ peptide.

## Conclusion

Brain overgrowth is a consistent endophenotype in 20% of individuals with autism. MRI volumetric studies showed overgrowth for both gray and white matter for young children (ages 2–6) with autism, coincidental to presentation of autistic symptoms. The trajectory of brain growth slows in adolescence and may show decreased growth at older ages. Enlargement of brain matter in autism may be due to a combination of elevated metabolic processes, migrational abnormality, and/or neuroinflammation. Recognizing potential contribution of the non-amyloidogenic pathway of βAPP processing to brain enlargement in autism enables novel adaptation of long-known AD pathway analyses to autism. Increased sAPPα and the ADAM family α-secretases may directly increase oligodendrocyte myelination or the neuroinflammatory response that promotes axonal sprouting of neurons and astrocyte activation. Consequently sAPPα and the ADAM family α-secretases, activated ERK receptor signaling, can activate P13K/AKt/mTOR. Resulting activation of Rho GTPases would favor OPC stimulation, thus enhancing myelination by activation of cofilin. Identification of new roles for AD pathways in autism may lead to new treatments for this enigmatic disorder.

## Author Contributions

All authors listed have made a substantial, direct and intellectual contribution to the work, and approved it for publication.

### Conflict of Interest Statement

DL is a member of the advisory boards for Entia Biosciences, Drug Discovery and Therapy World Congress, and Provaidya LLC. He also has stock options from QR Pharma for patents or patents pending on AIT-082, Memantine, Acamprosate, and GILZ analogs. DL also had prior funding from Baxter and Forest Research Labs. The remaining authors declare that the research was conducted in the absence of any commercial or financial relationships that could be construed as a potential conflict of interest.
